# Circadian rhythm of heart rate and heart rate variability in pregnancy

**DOI:** 10.1038/s44294-025-00107-6

**Published:** 2025-10-07

**Authors:** Mahkameh Rasouli, Mohammad Feli, Iman Azimi, Shahab Haghayegh, Fatemeh Sarhaddi, Hannakaisa Niela-Vilen, Anna Axelin, Pasi Liljeberg, Amir M. Rahmani

**Affiliations:** 1 School of Nursing, University of California, Irvine, CA USA; 2 Department of Computer Science, University of California, Irvine, CA USA; 3 Department of Computing, University of Turku, Turku, Finland; 4 Department of Anesthesia, Critical Care and Pain Medicine, Massachusetts General Hospital, Boston, MA USA; 5 Harvard Medical School, Boston, MA USA; 6 Broad Institute, Cambridge, MA USA; 7 Department of computer science, University of Helsinki, Helsinki, Finland; 8 Department of Nursing Science, University of Turku, Turku, Finland

**Keywords:** Physiology, Reproductive biology, Reproductive disorders

## Abstract

The autonomic nervous system (ANS) regulates physiological changes during pregnancy, supporting fetal development and homeostasis. Heart rate (HR) and heart rate variability (HRV) are well-established, non-invasive biomarkers of ANS function. However, their circadian dynamics during pregnancy remain under-explored due to the lack of continuous data collection, a gap now addressed by wearable technology. This study is the first comprehensive investigation of HR and HRV circadian rhythms throughout pregnancy using wearable devices in a free-living setting. We extract longitudinal HR and HRV from smartwatch Photoplethysmography (PPG) data via a machine learning-based pipeline and employ Cosinor analysis to assess circadian rhythm characteristics. Our findings revealed significant HR and HRV circadian patterns over 16 weeks, showing a decline in HRV, an increase in HR rhythm-adjusted mean, and elevated nighttime stress linked to sleep disturbances and daytime fatigue. These results offer valuable insights into ANS regulation during pregnancy and highlight the potential of HR and HRV circadian rhythm parameters as biomarkers for pregnancy complications.

## Introduction

Pregnancy is a critical period during which the autonomic nervous system (ANS) plays a vital role in regulating biological functions and maintaining homeostasis to support fetal development^1,2^. Evaluating ANS function through the changing patterns of heart rate (HR) and heart rate variability (HRV), widely recognized as a noninvasive indicator of ANS activity^3^, offers valuable insights into the physiological adaptations occurring during pregnancy^4,5^. The patterns of change in HR^6^ and HRV^7^ follow the circadian rhythm, which has approximately a 24-h cycle and is among the most well-known biological rhythms^8^.

**Fig. 1:**
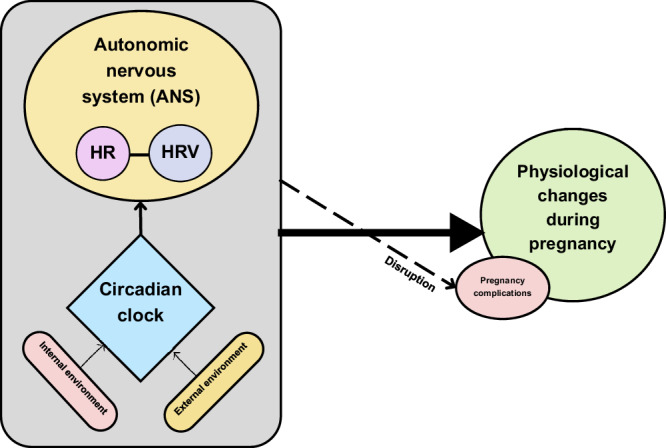
Conceptual framework. Exploring heart rate (HR) and heart rate variability (HRV) circadian rhythms during pregnancy and their association with pregnancy complications.

Circadian rhythms are regulated by central and peripheral clocks and influenced by photic cues, such as light exposure, as well as non-photic cues, like the sleep-wake cycle, meal timing, body temperature, and physical activity^9^. Disruptions in HR and HRV circadian rhythms have been linked to various disorders^10,11^, underscoring the potential for timing-specific treatments and targeting abnormal circadian rhythms as a complementary approach to standard care practices for certain pathologies. Therefore, understanding the circadian patterns of HR and HRV during pregnancy provides valuable insights into the regulation of the ANS in response to both photic and non-photic cues and could contribute to understanding the underlying causes of pregnancy complications, such as preterm birth^12–15^, gestational diabetes^16,17^, gestational hypertension^18^, pre-eclampsia^19–21^, and eclampsia^22,23^, for which few or no strong etiological factors have been firmly established. The conceptual framework representing these relationships is illustrated in Fig. 1.

There are few studies specifically investigating the circadian rhythms of HR and HRV during pregnancy. Some studies have utilized ambulatory blood pressure monitoring (ABPM) devices to capture episodic data in controlled environments, reporting only changes in HR rhythm-adjusted mean values^24,25^. Others have employed Electrocardiogram (ECG) devices^26–29^, allowing for the measurement of HR and frequency domain HRV metrics. Notably, Ekholm et al.^26^ conducted the first known study examining HRV circadian rhythms in pregnancy using Holter ECG, which enabled data collection during participants’ normal daily activities and sleep-wake cycles. Their findings indicated that all components of HRV power spectral analysis exhibited circadian rhythms, although robust statistical evidence for the presence of these rhythms was not provided. Another remarkable study involved participants in a free-living environment, adhering to strict protocols for sleep-wake cycles, diet, and physical activities. This study was the first to statistically confirm the presence of circadian rhythms in HRV changes during the first trimester of pregnancy, utilizing the Cosinor method^29^. Furthuremore, studies such as those by Stein et al.^28^, have not only explored the circadian rhythm of HRV but also investigated its deviations in complicated pregnancies, which potentially may aid in the identification of pregnancies at risk in the early weeks.

However, a significant limitation of previous studies is the lack of long-term, continuous data collection in free-living environments. Devices like ABPM and ECG, including Holter ECG, provide only short-term and non-continuous assessments due to the challenges of prolonged lab stays or extended periods of wearing these devices. This episodic approach falls short in capturing the dynamic nature of pregnancy adaptations and their circadian rhythms throughout all stages, thereby limiting our understanding of when, where, and how ANS alterations occur during pregnancy and their predictive value for pregnancy outcomes. A critical gap in the literature is the absence of studies examining circadian rhythms throughout all stages of pregnancy due to the lack of long-term, continuous monitoring. While Curione et al.^29^ confirmed the presence of circadian rhythms in HR and HRV changes during the first trimester, there is a notable lack of research on the existence and characteristics of circadian rhythms during the second and third trimesters, periods marked by significant physiological changes and an increased likelihood of complications.

Recent advancements in wearables for health monitoring have enabled continuous, cost-effective, and real-time data collection throughout pregnancy, including HR and HRV measurements. Devices such as the Oura ring (ouraring.com), Samsung Galaxy Watch (samsung.com/us/watches/), and Fitbit Sense (fitbit.com/sg/sense) allow long-term monitoring in free-living settings, addressing the limitations of episodic data collection^14,30–35^. These wearables offer dual benefits of participant comfort and the ability to monitor lifestyle factors such as sleep-wake cycles, diet, and physical activities without requiring restrictive protocols^36^. Additionally, advances in machine learning have improved the processing of wearable Photoplethysmogram (PPG) signals—an optical technique that measures blood volume changes to estimate cardiovascular metrics-enabling the extraction of reliable HR and HRV measurements. Therefore, the advancement in wearable technology and computing offers the opportunity to continuously collect HR and HRV data throughout pregnancy, potentially extending into the perinatal and postpartum phases, facilitating the investigation of circadian rhythms in real-world conditions, an area that remains largely understudied.

This study presents the first comprehensive investigation of circadian rhythms of HR and HRV throughout pregnancy, with a particular focus on the second and third trimesters. The primary aim was to examine the presence and temporal patterns of these rhythms in low-risk pregnancies. We hypothesized that HR and HRV would exhibit statistically significant circadian rhythms during these stages. Leveraging continuous, longitudinal data collection in a free-living setting using wearable devices, we developed machine-learning models to extract HR and HRV from PPG signals through a reliable processing pipeline and impute missing data. Additionally, Cosinor statistical analysis^37^ was applied to assess the presence and characteristics of circadian rhythms in HR and HRV. By establishing normative patterns, this study enhances our understanding of autonomic adaptations during pregnancy, enables future efforts toward early detection of pregnancy-related complications, and contributes to the development of personalized prenatal care strategies.

## Results

### Overview

In this study, we explored the circadian rhythms of HR and HRV in healthy pregnant women. Analyzing these rhythms provides valuable insights into the physiological adaptations during pregnancy and potential risks to maternal and fetal well-being. To achieve this, we collected continuous, longitudinal HR and HRV data from pregnant women in free-living conditions. We applied Cosinor analysis, a method specifically designed for modeling rhythmic biological processes, to interpret these patterns.

### Population characteristics

We describe the study population to provide context for our circadian rhythm analysis results. Our study included data from 30 pregnant women monitored during their second and third trimesters. The participants were recruited at a mean gestational age of 14 weeks and followed through delivery, with a mean gestational age at delivery of 39.8 weeks. Participants had an average age of 31.5 years, ranging from 23 to 39 years. All women had low-risk pregnancy profiles, defined as having a history of full-term births (i.e., after gestational week 37) and no prior pregnancy losses or complications. We continuously collected data using Samsung Gear Sport smartwatches (samsung.com/us/mobile/wearables/smartwatches/gear-sport-black-sm-r600nzkaxar/) in free-living conditions, recording HR and HRV measurements during both sleep and daily activities. A detailed summary of population characteristics can be found in Table 1.

**Table 1:** Participants’ background information

Characteristic	(*n* = 30)
Age, mean (range)	31.5 (23–39)
Body mass index (BMI), mean (range)	25.1 (17.8–45.9)
Married/cohabiting, *n* (%)	30 (100)
Employed/full-time student, *n* (%)	28 (93)
University level education, *n* (%)	8 (27)
Pregnancy planned, *n* (%)	27 (90)
Gestational age at recruitment (week + day), mean (SD)	14 + 2.8 (0.8 + 1.3)
Gestational age at delivery (week + day), mean (SD)	39.8 + 2.7 (1.3 + 1.9)

*SD* stands for standard deviation.

### HR circadian rhythm

In this section, we present the results of our analysis of HR circadian rhythms during pregnancy. First, we provide the overall rhythmicity parameters for all pregnancy weeks in our study, examining trends in key rhythmicity metrics. Following this, we demonstrate a more detailed analysis of HR circadian rhythm patterns for specific pregnancy weeks.

We conducted a detailed analysis of HR circadian rhythms for all individuals across various pregnancy weeks. The Cosinor model was first applied to each participant’s daily 24-h HR data. Then, the weekly averages for each participant were calculated by averaging the daily Cosinor models across different days of the week. Finally, the population’s overall HR rhythmic pattern for each week was derived as the mean of the individual participants’ weekly averages.

The Cosinor analysis examined three key rhythmicity parameters: amplitude, Midline Statistic of Rhythm (MESOR), and acrophase. Amplitude refers to half the distance between the maximum and minimum values of the rhythm, indicating the intensity of fluctuations. MESOR represents the rhythm-adjusted mean around which fluctuations occur, serving as the baseline. The acrophase indicates the timing of the peak in the rhythm. In addition to these parameters, we report *p* values that assess the statistical significance of the detected 24-h rhythmicity. These *p* values test the null hypothesis that no circadian pattern is present in the aggregated data for a specific pregnancy week. A *p* value below 0.05 indicates statistically significant evidence of a 24-h rhythm. The *p* values along with residual standard error (SE) for each weekly population-level Cosinor fit of HR are presented in the Supplementary Table 1.

**Fig. 2:**
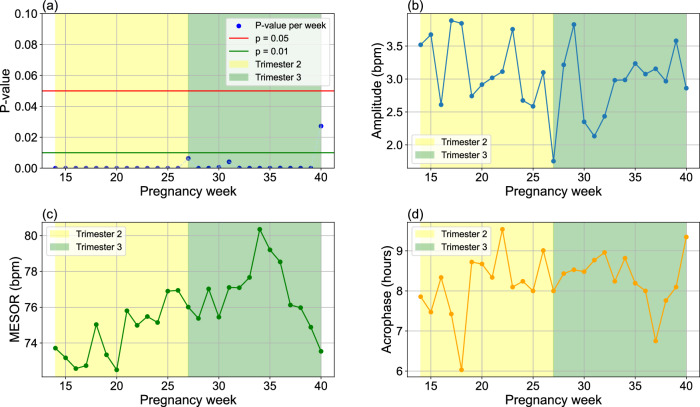
Cosinor analysis of HR circadian rhythms during pregnancy. Trend of **a** *p* values, **b** amplitude, **c** midline estimating statistic of rhythm (MESOR), and **d** acrophase changes from gestational week 14–40. The MESOR and amplitude are reported in beats per minute (bpm), and acrophase is reported as clock time in hours. A *p* value of <0.05 is considered statistically significant.

Figure 2 demonstrates the overall *p* values, amplitude, MESOR, and acrophase of HR circadian rhythms for pregnancy weeks 14–40. As illustrated in Fig. 2a, the analysis from gestational weeks 14–39 shows highly significant *p* values, all below the 0.01 threshold. Week 40 also shows statistical significance, with a *p* value below 0.05, indicating robust circadian patterns in HR.

Figure 2b illustrates the amplitude of the HR circadian rhythms for weeks 14–40, covering the second trimester (14–27 weeks) and third trimester (27–40 weeks). As indicated, a downward trend is observed during the second trimester, starting at 3.5 BPM in week 14 and decreasing to less than 2 BPM by week 27. In contrast, an upward trend is observed in the third trimester, reaching about 3 BPM by week 40. These trends indicate that the intensity of the HR circadian rhythm decreases during the second trimester but gradually increases as pregnancy advances into the third trimester.

In addition, Fig. 2c illustrates the MESOR from the Cosinor analysis of HR circadian rhythms. A significant upward trend is observed from week 14 to week 34, starting at approximately 74 BPM and rising to around 80 BPM. Following this, a highly significant downward trend emerges from week 34 to week 40, bringing the MESOR below 74 BPM by the end of the period. These findings show a shifting circadian rhythm baseline in HR, with an increase during the second trimester and a decline in the later weeks of the third pregnancy trimester.

Figure 2d presents the acrophase of the HR circadian rhythms. As shown, no significant trend was detected in the acrophase across all weeks, indicating minimal change over time. This suggests that, overall, the timing of the peaks in HR circadian rhythms remained relatively stable throughout the pregnancy period.

**Fig. 3:**
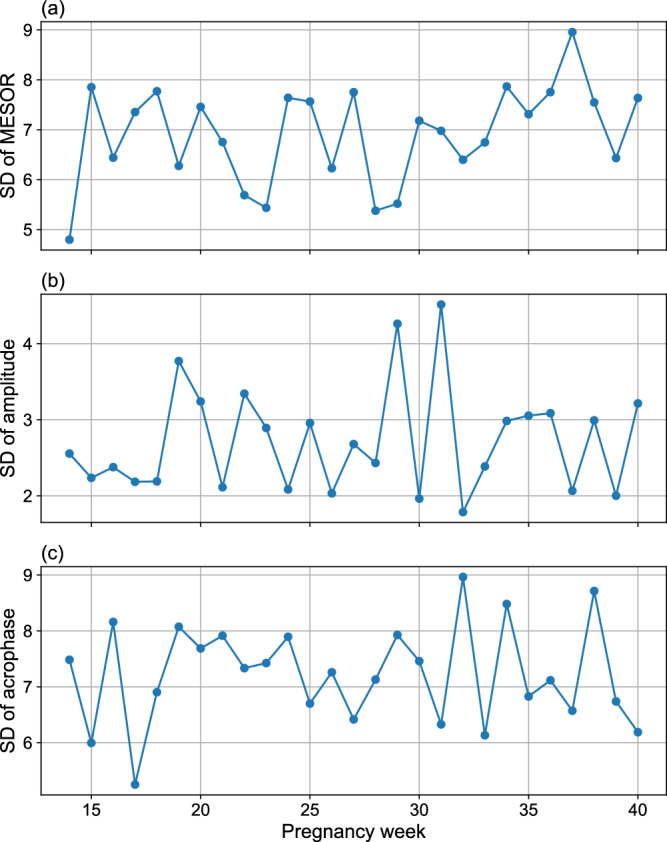
Inter-individual variation in HR rhythmic parameters. The plots show the standard deviation (SD) across participants for **a** MESOR, **b** amplitude, and **c** acrophase of HR from gestational weeks 14–40.

Furthermore, we quantified inter-individual variability in HR circadian parameters across pregnancy weeks. As shown in Fig. 3, the MESOR standard deviation remained relatively stable throughout pregnancy, averaging around 7 bpm, with a slight increase toward late gestation, peaking at week 39 (around 9 bpm). The amplitude variation was lower than MESOR but fluctuated across weeks, with the highest inter-individual difference (about 4.6 bpm) observed around week 32. Acrophase inter-individual variation was approximately 7–8 h, suggesting moderate heterogeneity in circadian phase alignment across participants.

**Fig. 4:**
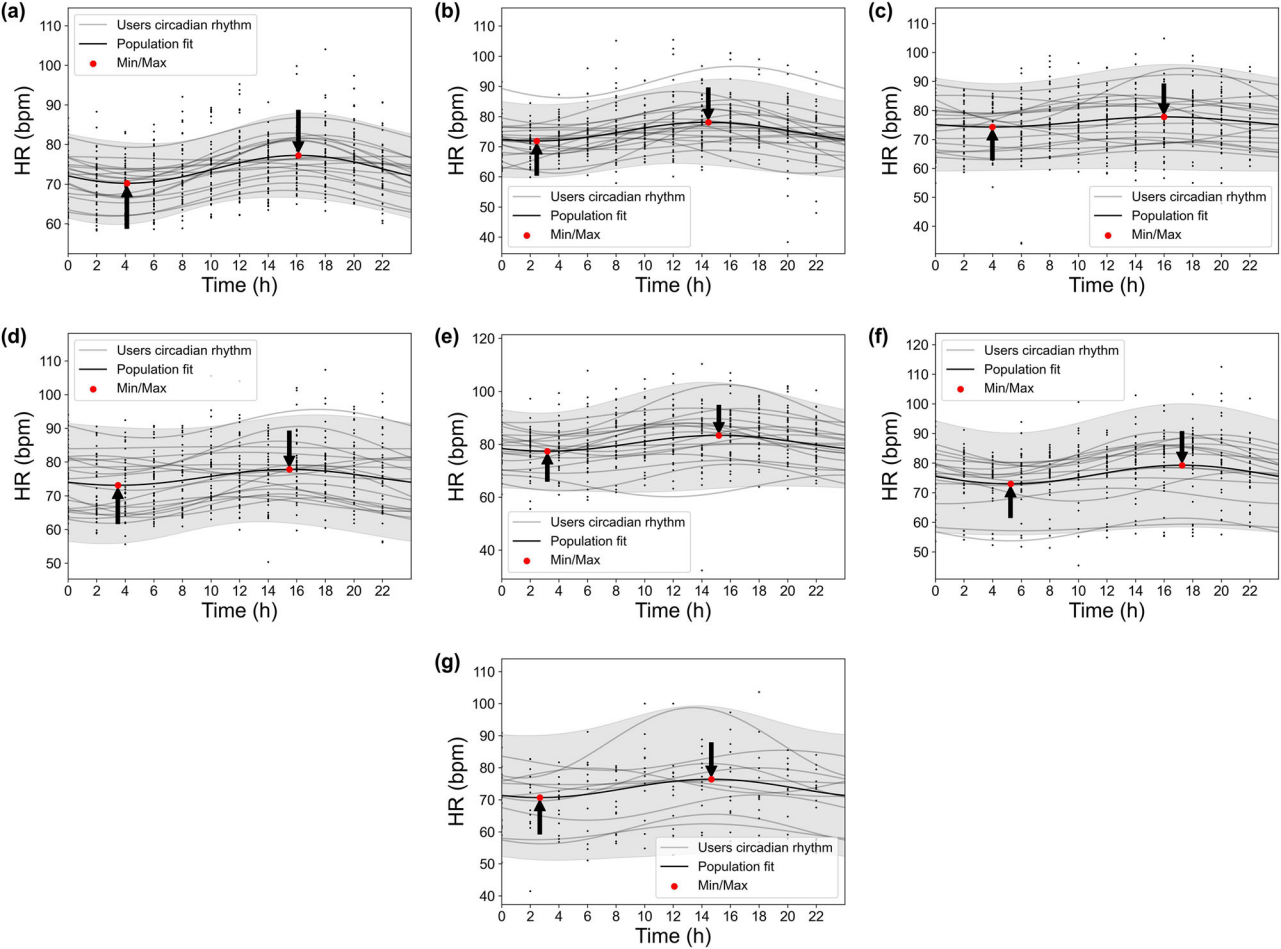
Circadian rhythms of HR over 24 h across different pregnancy weeks. Each subplot corresponds to a different gestational week: **a** Week 14, **b** Week 22, **c** Week 27, **d** Week 30, **e** Week 34, **f** Week 37, and **g** Week 40. Black dots represent individual participants' weekly-averaged HR; gray lines indicate their corresponding fitted circadian rhythm. The thick black line denotes the population-level mean circadian rhythm, and the gray shaded region represents the 95% confidence interval of the population-level fitted circadian rhythm. All weeks shown exhibited statistically significant rhythmicity (*p* < 0.05).

We present the detailed Cosinor analysis results of HR circadian rhythms across pregnancy weeks 14, 22, 27, 30, 34, 37, and 40 (Fig. 4). Weeks 14 and 40 were the first and last weeks in our analysis, while week 27 represents the end of the second trimester. Weeks 30, 34, and 37 also revealed significant findings. It should be noted that further HR circadian rhythm analyses for other pregnancy weeks are included in the Supplementary Fig. 1 for reference.

In Fig. 4, black dots and gray lines represent the weekly-averaged circadian data and rhythms for individual participants, respectively. The black lines show the population-mean model, highlighting the overall rhythmic pattern for the entire population. The gray-shaded regions represent the 95% confidence interval of the population-level fitted circadian rhythms. As indicated, analysis of the population-mean HR revealed systematic changes in both timing and magnitude of daily minima and maxima across gestation. It should be noted that the number of data points (black dots) varies across weeks due to the unavailability of data from certain participants during specific weeks.

In week 14, HR exhibited a minimum of around 70 bpm at 04:00 and reached its maximum of just below 80 bpm at 16:00. By week 22, both the timing and magnitude shifted, with the minimum of 70 bpm occurring earlier at 02:00 and the maximum decreasing to around 75 bpm at 14:00. At week 27, the amplitude of oscillation diminished substantially, with both minimum and maximum values converging at 75 bpm, occurring at 04:00 and 16:00 respectively. Week 30 demonstrated patterns similar to week 27, though with slightly advanced timing. A notable increase in HR values was observed at week 34, with the minimum rising to 80 bpm at 03:00 and the maximum exceeding 80 bpm at 15:00. During week 37, the timing of extrema showed the greatest delay, with minimum and maximum values (both ranging 70–80 bpm) occurring at 05:00 and 17:00, respectively. Finally, by week 40, the pattern shifted earlier again, with the minimum decreasing to 70 bpm after 02:00 and the maximum reaching around 75 bpm after 14:00.

### HRV circadian rhythm

This section presents the findings of our analysis of HRV circadian rhythms, focusing on the root mean square of successive differences (RMSSD) between heartbeats. RMSSD is a crucial parameter indicating the variability in time between consecutive heartbeats. We analyzed both overall HRV rhythmicity parameters throughout pregnancy and HRV circadian rhythms at specific pregnancy weeks.

**Fig. 5:**
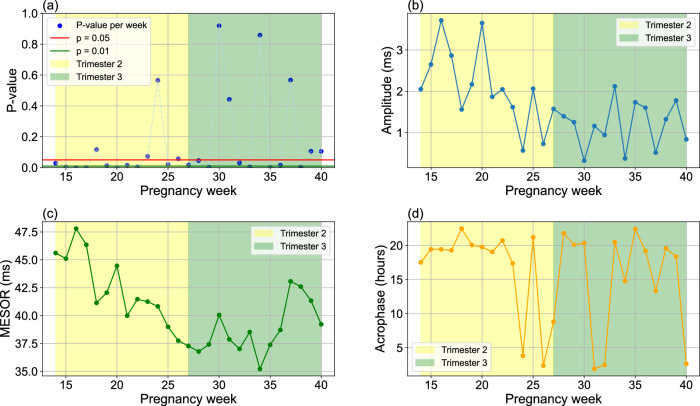
Cosinor analysis of HRV circadian rhythms during pregnancy. Trend of **a** *p* values, **b** amplitude, **c** midline estimating statistic of rhythm (MESOR), and **d** acrophase changes in the root mean square of successive differences (RMSSD), a HRV time-domain measure, from gestational week 14–40. The MESOR and amplitude are reported in milliseconds (ms), and acrophase is reported as clock time in hours. A *p* value of <0.05 is considered statistically significant.

We analyzed HRV circadian rhythms for all individuals across various pregnancy weeks. Similar to our approach used for HR analysis, the Cosinor model was applied separately to each participant’s daily 24-h HRV data. Weekly averages for each participant were then calculated by averaging the daily Cosinor models across several days of the week. Finally, the population’s overall HRV rhythmic pattern for each week was determined as the mean of the individual participants’ weekly averages. These results, including temporal evolution of *p* values, amplitude, MESOR, and acrophase across pregnancy weeks 14–40, are summarized in Fig. 5. The *p* values along with residual SE for each weekly population-level Cosinor fit of HRV are presented in the Supplementary Table 2.

As shown in Fig. 5a, analysis of RMSSD demonstrated statistically significant circadian patterns (*p* < 0.05) in most pregnancy weeks. Figure 5b illustrates the amplitude of RMSSD circadian rhythms throughout pregnancy. The results show a consistent downward trend across both the second and third trimesters-beginning above 2 ms in week 14 and declining to less than 1 ms by week 40-indicating a progressive weakening in the strength of HRV rhythmic fluctuations as gestation advances.

The MESOR analysis (Fig. 5c) identified a significant downward trend from week 14 to week 34, with values declining from approximately 46 ms to below 36 ms. Following this decline, a transient increase was observed for MESOR during weeks 35–37, reaching approximately 43 ms, before decreasing again in the final weeks of the third trimester to around 39 ms.

As illustrated in Fig. 5d, no significant trends were observed in the acrophase values of RMSSD. However, the acrophase for most pregnancy weeks occurred after 15:00, indicating that the peaks of HRV rhythms were generally concentrated in the afternoon.

**Fig. 6:**
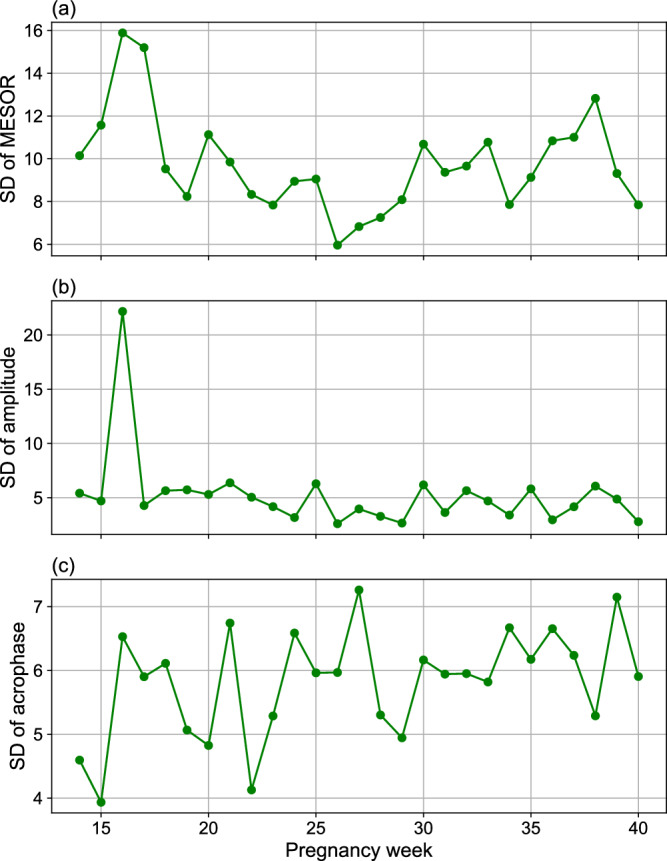
Inter-individual variation in HRV rhythmic parameters. The plots show the standard deviation (SD) across participants for **a** MESOR, **b** amplitude, and **c** acrophase of RMSSD from gestational weeks 14–40.

Figure 6 shows the inter-individual standard deviations in HRV (RMSSD) rhythmic parameters. MESOR showed the highest variability in early pregnancy (weeks 15–17), peaking at approximately 16 ms, then gradually declining and stabilizing during mid to late pregnancy (around 8–12 ms). Amplitude variability displayed a sharp peak at week 16 (about 22 ms), then remained moderate and stable (around 5 ms). Acrophase variation fluctuates around 4–7 h across all weeks.

**Fig. 7:**
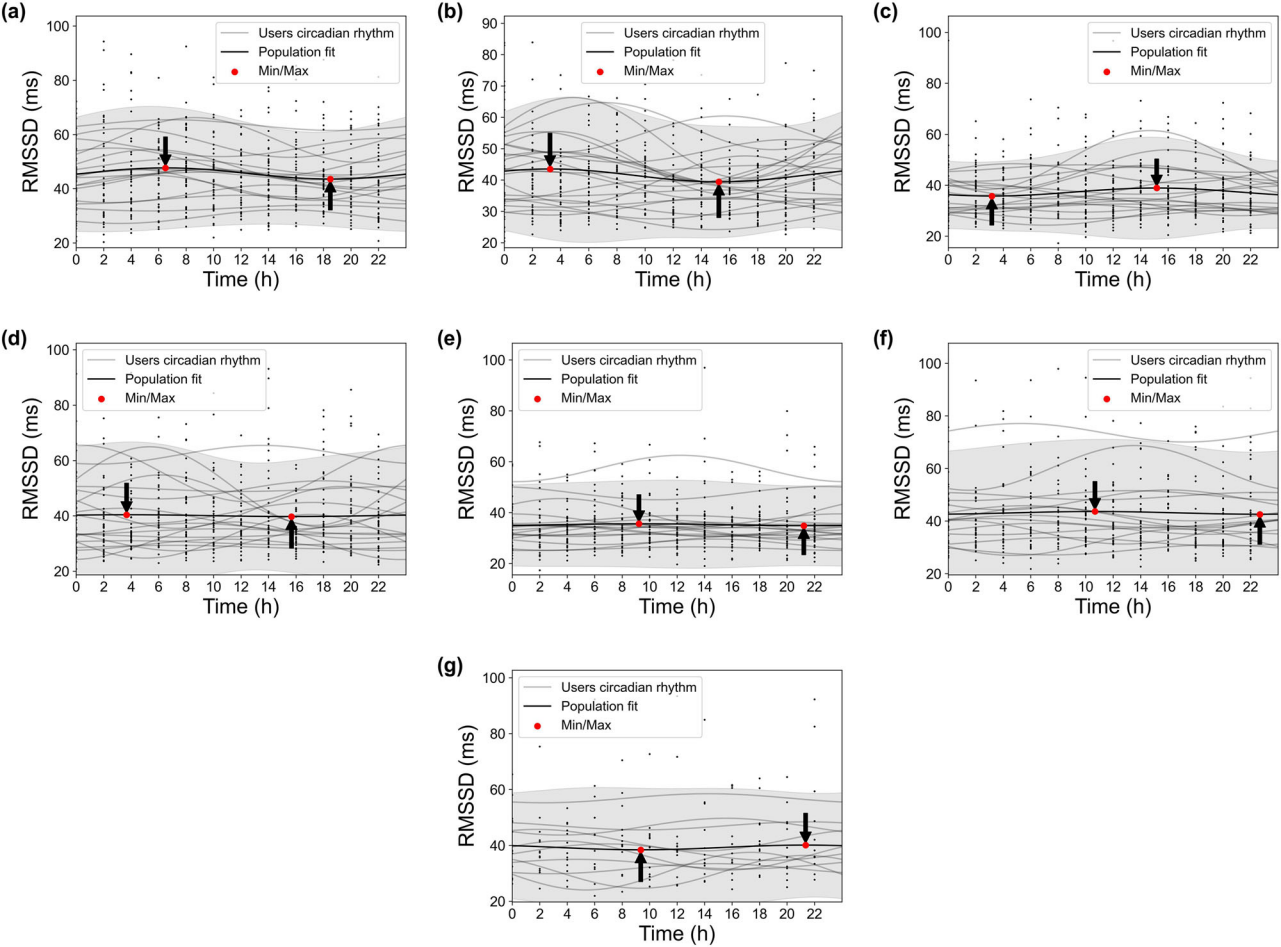
Circadian rhythms of HRV over 24 h across different pregnancy weeks. Each subplot corresponds to a different gestational week: **a** Week 14 (*p* < 0.05), **b** Week 22 (*p* < 0.05), **c** Week 27 (*p* < 0.05), **d** Week 30 (*p* = 0.919), **e** Week 34 (*p* = 0.859), **f** Week 37 (*p* = 0.567), and **g** Week 40 (*p* = 0.106). Black dots represent individual participants' weekly-averaged HRV; gray lines indicate their corresponding fitted circadian rhythm. The thick black line denotes the population-level mean circadian rhythm, and the gray shaded region represents the 95% confidence interval of the population-level fitted circadian rhythm.

The results of Cosinor analysis of RMSSD for pregnancy weeks 14, 22, 27, 30, 34, 37, and 40 are demonstrated in Fig. 7. The analysis revealed significant circadian variations in RMSSD for weeks 14, 22, and 27 (*p* < 0.05), while the patterns for weeks 30, 34, 37, and 40 did not meet the significance threshold (*p* > 0.05). Additional analyses of HRV circadian rhythms for other pregnancy weeks are provided in the Supplementary Fig. 2 for further reference.

In week 14, the maximum RMSSD occurred around 6:00, with values slightly below 50 ms, while the minimum was observed just after 18:00, at approximately 45 ms. By week 22, the maximum RMSSD shifted to around 3:00 AM, exceeding 40 ms, and the minimum appeared just after 15:00, reaching about 40 ms. Interestingly, in week 27, the pattern was inverted compared to previous weeks, with the minimum RMSSD occurring at around 3:00 (below 40 ms) and the maximum observed slightly after 15:00 (~40 ms). Although this inversion was statistically significant at the group level, a more detailed inspection of individual-level acrophases revealed substantial inter-individual variability, with some participants maintaining earlier peaks. These results are illustrated in a polar plot in the Supplementary Fig. 3, highlighting the non-uniform nature of the phase shift across all individuals.

For weeks 30, 34, 37, and 40, the analysis did not reveal significant circadian variations, as both the maximum and minimum RMSSD values hovered around 40 ms. No notable differences were detected between day and night for the population-mean model during these weeks, indicating a less prominent oscillatory pattern.

**Fig. 8:**
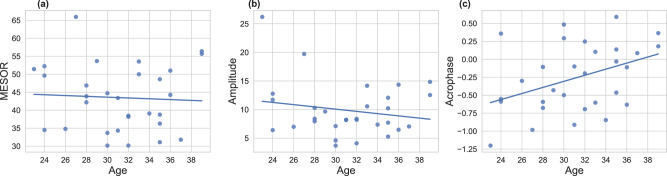
Correlation between participant age and circadian rhythm parameters of HRV. MESOR and Amplitude are expressed in ms, and acrophase is reported in radians (rad). **a** MESOR vs. age (*r* = −0.05, *p* = 0.775); **b** amplitude vs. age (*r* = −0.19, *p* = 0.324); **c** acrophase vs. age (*r* = 0.40, *p* = 0.029).

To explore the potential influence of age on circadian rhythm characteristics of HRV during pregnancy, we analyzed the correlation between participants’ age and the mean values of MESOR, amplitude, and acrophase of RMSSD across all pregnancy weeks. As shown in Fig. 8, no significant correlations were observed between age and MESOR (*r* = −0.05, *p* = 0.775) or amplitude (*r* = −0.19, *p* = 0.324). However, a moderate positive correlation was found between age and acrophase, expressed in radians (*r* = 0.40, *p* = 0.029), indicating that older participants tend to exhibit a later peak in HRV circadian rhythm.

We further examined circadian patterns of two additional HRV parameters: high-frequency (HF) power and pNN50-across pregnancy weeks. The corresponding rhythm parameters (MESOR, amplitude, acrophase, and *p* value) are summarized in Supplementary Figs. 4 and 5.

### Comparison of HR and HRV circadian rhythms in early versus late pregnancy

To further evaluate trends in circadian regulation, we compared HR and HRV rhythm parameters between early (weeks 14–20) and late pregnancy (weeks 34–40). As shown in Table 2, HR MESOR and amplitude increased significantly in late pregnancy compared to the early stages, suggesting an elevated baseline and modest enhancement in circadian modulation of HR. In contrast, HRV MESOR and amplitude significantly decreased as pregnancy progressed. Acrophase values for both HR and HRV showed no statistically significant shifts.

**Table 2:** Comparison of HR and HRV circadian rhythm parameters between early and late pregnancy

	Parameter	Early mean	Late mean	*p* value	Effect size
HR	MESOR (bpm)	73.15	77.11	0.0004	−0.35
	Ampl (bpm)	5.90	6.67	0.0405	−0.20
	Acro (rad)	0.68	0.67	0.9551	0.01
HRV	MESOR (ms)	44.89	40.21	0.0047	0.27
	Ampl (ms)	10.61	8.03	0.0319	0.21
	Acro (rad)	−0.35	−0.06	0.1893	−0.13

Early pregnancy is defined as gestational weeks 14–20, and late pregnancy as weeks 34–40. Values represent group-level means. MESOR and amplitude are reported in bpm, and acrophase is reported in radians (rad). Effect size is reported using Cohen’s d. A *p* value of <0.05 is considered statistically significant.

### Sensitivity analysis results

To evaluate the robustness and stability of our population-level circadian rhythm estimates across gestational weeks, we conducted a Leave-One-Out Cross-Validation (LOOCV) analysis. As presented in Table 3, the results demonstrated highly stable estimates for both HR and HRV. For HR, the average standard deviation across all weeks was 0.42 bpm for MESOR, 0.16 bpm for amplitude, and 0.24 h for acrophase, indicating stable rhythmic estimates with minimal sensitivity to individual participants. Similarly, for HRV, the average standard deviations were 0.60 ms for MESOR, 0.26 ms for amplitude, and around 1 h for acrophase.

**Table 3:** Summary of leave-one-out cross-validation (LOOCV) results for circadian parameters of HR and HRV

Week	HR			HRV (RMSSD)		
	MESOR	Amplitude	Acrophase	MESOR	Amplitude	Acrophase
14	73.55 (0.47)	3.62 (0.28)	7.31 (1.20)	47.40 (0.75)	4.38 (0.43)	18.24 (0.17)
15	73.13 (0.43)	3.79 (0.34)	7.16 (0.79)	45.81 (0.62)	2.92 (0.34)	18.42 (0.34)
16	72.57 (0.29)	2.61 (0.09)	8.32 (0.10)	49.63 (0.78)	3.78 (0.27)	19.82 (0.24)
17	72.72 (0.37)	3.92 (0.16)	7.34 (0.44)	47.19 (0.74)	3.97 (0.31)	19.22 (0.14)
18	75.03 (0.45)	3.85 (0.16)	6.01 (0.14)	41.34 (0.58)	1.59 (0.21)	22.19 (0.78)
19	73.34 (0.34)	2.74 (0.13)	8.71 (0.11)	42.86 (0.47)	2.62 (0.37)	19.81 (0.37)
20	72.50 (0.35)	2.92 (0.13)	8.65 (0.13)	45.21 (0.57)	3.92 (0.25)	18.79 (0.18)
21	75.80 (0.36)	3.02 (0.12)	8.35 (0.14)	40.85 (0.61)	1.64 (0.22)	21.21 (0.49)
22	74.99 (0.30)	3.11 (0.13)	9.56 (0.15)	41.61 (0.42)	2.16 (0.63)	21.66 (1.07)
23	75.48 (0.27)	3.76 (0.12)	8.10 (0.09)	41.80 (0.49)	2.26 (0.19)	17.45 (0.31)
24	75.15 (0.35)	2.68 (0.11)	8.24 (0.12)	40.83 (0.40)	0.59 (0.14)	3.75 (1.07)
25	76.90 (0.44)	2.59 (0.21)	7.97 (0.27)	39.10 (0.61)	2.27 (0.34)	21.20 (0.63)
26	76.94 (0.27)	3.10 (0.09)	8.99 (0.11)	37.75 (0.27)	0.74 (0.11)	2.41 (0.79)
27	76.01 (0.45)	1.76 (0.14)	8.02 (0.31)	37.28 (0.43)	1.58 (0.23)	8.82 (0.30)
28	75.37 (0.32)	3.22 (0.11)	8.45 (0.13)	36.99 (0.44)	1.57 (0.17)	21.66 (1.09)
29	77.02 (0.41)	3.83 (0.24)	8.54 (0.21)	37.51 (0.63)	1.29 (0.20)	20.63 (0.61)
30	75.45 (0.43)	2.35 (0.14)	8.50 (0.16)	40.65 (0.64)	1.30 (0.27)	22.90 (2.34)
31	77.10 (0.46)	2.14 (0.11)	8.77 (0.26)	38.13 (0.68)	1.60 (0.29)	2.38 (0.89)
32	77.09 (0.32)	2.43 (0.11)	8.97 (0.09)	37.65 (0.55)	1.00 (0.14)	4.81 (1.27)
33	77.66 (0.40)	2.98 (0.11)	8.22 (0.13)	38.53 (0.54)	2.13 (0.21)	20.51 (0.28)
34	80.35 (0.47)	2.99 (0.15)	8.79 (0.19)	35.22 (0.46)	0.40 (0.10)	16.31 (5.02)
35	79.20 (0.38)	3.24 (0.11)	8.18 (0.16)	37.50 (0.48)	1.85 (0.28)	21.99 (0.35)
36	78.53 (0.50)	3.08 (0.12)	7.99 (0.16)	38.82 (0.69)	1.69 (0.20)	19.64 (0.48)
37	76.12 (0.60)	3.16 (0.13)	6.75 (0.16)	44.02 (0.92)	1.26 (0.27)	14.53 (5.63)
38	75.97 (0.41)	2.97 (0.14)	7.74 (0.16)	42.74 (0.75)	1.47 (0.20)	19.76 (0.48)
39	74.88 (0.49)	3.58 (0.16)	8.11 (0.10)	41.64 (0.71)	2.08 (0.41)	19.18 (0.41)
40	73.54 (0.92)	2.87 (0.36)	9.32 (0.35)	39.36 (0.95)	1.16 (0.24)	2.42 (1.14)

Values are reported as mean (standard deviation) across gestational weeks. MESOR and amplitude are reported in beats per minute (bpm), and acrophase is reported in hours.

These results indicate that the circadian model is robust and not highly influenced by the exclusion of any single individual, confirming the stability of our population-level findings and providing quantitative evidence of the model’s resilience to inter-individual variability across the cohort.

## Discussion

Our study provides the first comprehensive investigation of HR and HRV circadian rhythms throughout pregnancy using continuous monitoring in free-living conditions. The key findings reveal: (1) the presence of statistically significant circadian patterns in both HR and HRV across pregnancy, and (2) distinct variations in these patterns associated with specific gestational weeks and trimesters. These findings have significant physiological implications, as the ANS plays a crucial role in regulating bodily functions and maintaining homeostasis during pregnancy to meet fetal requirements while protecting maternal health^1,2^. While previous studies have documented circadian rhythms in various physiological parameters, including HR and HRV in non-pregnant populations^6,7^, the examination of these rhythms during pregnancy has been limited due to methodological challenges in longitudinal data collection and analysis of physiological signals. Our study addresses these limitations by employing wearable technology for continuous data collection and utilizing advanced machine learning techniques for signal processing and imputation of missing data.

In this study, we employed Cosinor analysis, a widely used parametric method, to evaluate circadian rhythms in HR and HRV. Compared to non-parametric and frequency-domain approaches, Cosinor offers interpretable parameters-MESOR, amplitude, and acrophase-that are particularly suitable for assessing physiological patterns across pregnancy weeks. Aligned with the study’s aim and hypothesis, Cosinor enables statistical testing for the presence of circadian rhythms, rather than relying solely on visual inspection, and can model individual-level variability while also supporting population-level inference, accounting for each participant’s unique physiological characteristics instead of assuming population homogeneity. While Cosinor assumes a sinusoidal waveform and may be limited in detecting irregular or asymmetric rhythms, it is well-suited for analyzing physiological fluctuations in HR and HRV, which have been shown to follow sinusoidal patterns in non-pregnant individuals^38^ and during the first trimester of pregnancy^29^, evidence that informed our hypothesis. In contrast, alternative methods often lack intuitive parameters for temporal trend analysis and typically require categorical time inputs. Given our objective to investigate circadian rhythm parameters in HR and HRV across gestation using longitudinal data, Cosinor analysis provided a statistically rigorous and physiologically meaningful approach.

The findings of our study indicate a significant increasing trend in HR MESOR from week 14 to week 34, with the highest HR MESOR observed at week 34, followed by a pronounced decreasing pattern through the remainder of pregnancy (Fig. 2c). Figure 3 further shows a stable MESOR in HR across participants, with inter-individual standard deviations ranging from 4 to 9 beats per minute-peaking at week 37, which is within expected norms for healthy pregnancies^39^. In contrast, HRV MESOR declines significantly from week 14 and reaches its lowest point around week 34. Thereafter, it increases modestly, although it does not attain the levels observed at week 14 (Fig. 5c). Notably, week 16 marks the point of lowest HR MESOR and highest HRV MESOR (Figs. 4 and 5c). Figure 6 further demonstrates consistent MESOR across participants, with inter-individual variation ranging from approximately 6–16 ms, and the highest variability also observed at week 16. This population-level declining HRV trend can be attributed to the compensatory increase in heart rate required to meet the elevated cardiac output (CO) demands^40,41^, as well as the rising stress levels during pregnancy^42^. To further isolate the effects of stress on the ANS from the changes in HR due to increased CO demands, we analyzed the circadian rhythm of normalized HRV metrics^43,44^, which confirmed the same pattern. As a result, we conclude that the HRV decrease is due to the ANS stress, which peaks around week 34, shown in Fig. 5c. Given that MESOR represents the rhythm-adjusted mean for both HR and HRV, their patterns align closely with the mean HR and HRV reported in prior longitudinal studies conducted by Rowan et al.^30^ and sarhaddi et al.^34^. However, an important distinction from the study by Rowan et al.^30^ is that while they focused specifically on resting HR, which is typically lower, we measured HR throughout the entire day, resulting in higher values. Our approach provides the advantage of assessing HR and HRV during various daily activities and stressors, while other studies often rely solely on resting data.

The acrophase, described as the time of the amplitude/peak of HR, overall shows a delaying trend from the beginning of the second trimester (T2) to the end of pregnancy (Fig. 2d), despite small fluctuations. This overall trend suggests that the time at which the maximum HR occurs shifts progressively later in the day as pregnancy progresses. The HR acrophase reaches its lowest point in week 18 and its highest point in week 22. Despite the overall trajectory observed, there is notable inter-individual variability in acrophase timing across gestation, ranging from 5 to 9 h. This variability may reflect differences in chronotype, age, and lifestyle factors—especially those related to daylight exposure and sleep disturbances. However, sensitivity analysis indicates that our circadian rhythm analysis is not significantly influenced by individual participant characteristics; eliminating a participant results in an average change of only 0.24 h in acrophase timing. A more detailed analysis of HR acrophase reveals a complex, heterogeneous pattern that extends beyond a simple linear trend.

The HRV acrophase shows greater fluctuations after week 23 (Fig. 5d), along with substantial inter-individual variability across most weeks (Fig. 6), similarly observed in HR acrophase timing. An intriguing finding emerges in week 27, where the lowest HRV, which typically occurs in the evening due to daily stress, shifts to become the peak, suggesting a less stressful period during that time of day (Fig. 7). Conversely, the minimum HRV occurs around 3 a.m. in week 27, a time traditionally associated with reduced stress due to sleep and rest (Fig. 7). This “reversed plot” suggests increased stress levels during sleep, which may contribute to or result from sleep disturbances. These findings align with previous research on the declining quality of sleep, as measured by the Pittsburgh Sleep Quality Index^45,46^, which begins to decline at the start of the third trimester compared to the second, despite a significant increase in total sleep duration^47^. This may explain why restless nights contribute to increased daytime fatigue and a greater need for afternoon naps. Indeed, a study shows a decrease in average nighttime sleep, an increase in the number of nighttime awakenings during months 7 and 8, and, finally, a higher frequency of daytime naps per week in month 8 and beyond compared to the average for the entire pregnancy^48^. These sleep disturbances are attributed to factors such as reflux, leg cramps, frequent urination, back pain, hip/pelvic discomfort, difficulty finding a comfortable position, baby movements, contractions, and concerns about delivery, all of which become significantly more common during the third trimester compared to the second^48^. While the reversed plot in week 27 is statistically significant (*p* < 0.05) at the group level (Supplementary Table 2), reflecting physiological adaptation within the pregnant population, our individual-level analysis revealed that this reversal was not consistently observed across all participants’ circadian profiles (Supplementary Fig. 3). Additionally, the inter-individual variation in HRV acrophase during week 27 was the highest among all weeks, with an average difference of approximately 7 h between individuals (Fig. 6). This variability highlights how individual differences in lifestyle factors, age, chronotype, sleep patterns, and the prevalence of pregnancy-related sleep disturbances may influence HRV acrophase. Despite these variations, our sensitivity analysis shows that the circadian rhythms of HRV parameters are not substantially affected by participant-level differences, with an average change of only about 1 h in acrophase when excluding a participant and just 0.30 h on average for week 27 (Table 3).

The HRV amplitude, defined as the difference between peak and trough (highest and lowest values, respectively), shows an overall decreasing trend throughout the second and third trimesters (Fig. 5b), indicating that the circadian rhythm of HRV becomes flatter as pregnancy progresses. Inter-individual variability in HRV amplitude remains largely stable throughout pregnancy, with a notable spike observed around week 16 (Fig. 6), the week that also exhibits the highest HRV amplitude across all weeks (Fig. 5b). In certain weeks, such as week 30 (Figs. 5b and 7), the amplitude is particularly low, making it challenging to visually distinguish peaks and troughs. This reduced curvature in the HRV circadian rhythm can be attributed to an overall increase in stress levels compared to the early weeks of the second trimester, particularly elevated nighttime stress in later weeks, as the circadian rhythms in Fig. 7 show more pronounced changes in the nighttime curve. These stress-related factors lead to reduced variation in RMSSD, often as a result of sleep disturbances. This suggests that elevated stress levels disproportionately affect nighttime and sleep more than daytime during weeks with low amplitude, as shown in the circadian rhythms in Fig. 7.

Conversely, HR amplitude decreases from week 14 to week 27, after which it begins to rise toward the end of pregnancy (Fig. 2b). The inter-individual variability in HR amplitude remains consistently low throughout pregnancy, with differences ranging between approximately 1 and 5 beats per minutes (Fig. 3). In week 27, the lowest amplitude is observed, with minimal variation between daytime and nighttime HR (Fig. 4), which can be attributed to the influence of sleep disturbances. Additionally, as mentioned earlier, the HRV acrophase (timing of HRV amplitude) in week 27 indicates that the HRV trough occurs during nighttime, based on the circadian rhythms in Fig. 5d, resulting in a reversed circadian pattern. While week 27 may not be the most stressful week of pregnancy, it exhibits the least HR variation (Figs. 2b and 4), with the HRV trough occurring at night.

Our correlation analysis examining the relationship between maternal age and circadian rhythm characteristics of HRV across all pregnancy weeks revealed no significant association between age and HRV MESOR or amplitude. This finding aligns with previous literature in non-pregnant (male and female) healthy populations, which reported no significant differences in RMSSD values between age groups 20–29 and 30–39 years—comparable to the age distribution in our sample^49^. However, we observed a moderate correlation between age and HRV acrophase, indicating that older participants tended to exhibit a delayed peak in HRV rhythm. This observation is consistent with prior studies in non-pregnant populations, where a 10-year increase in age was associated with approximately a 1 h and 10 min delay in RMSSD acrophase^50^. These findings may help explain the high inter-individual variability in HRV acrophase observed in our study, given our participants’ age range of 23–39 years. However, the variability in our data exceeded the expected delay reported in previous studies, suggesting that additional confounding factors or pregnancy-specific modulations may also contribute to this variation.

Despite individual lifestyle variations, our inter-individual variation analysis for both HR and HRV revealed robust circadian MESOR and amplitude across the population, with the exception of acrophase, which exhibited relatively high standard deviation in both HR and HRV. However, sensitivity analysis showed that removing individual participants resulted in mean changes of only about one hour in acrophase estimates, suggesting that this variability did not compromise the robustness of the circadian rhythm findings. Additionally, no significant difference in acrophase levels was observed between early and late pregnancy, and no consistent trend emerged for acrophase in either HR or HRV. Furthermore, age appeared to influence HRV acrophase. These observations suggest that the elevated standard deviation in acrophase may reflect normal inter-individual variation within this population rather than introducing bias into the analysis. Inter-individual behavioral differences may be the primary contributors to this variability, warranting further research to determine whether such variation is typical and to identify the behavioral or physiological factors driving these differences and their directional effects.

To simplify the overall trajectory of HR and HRV circadian dynamics between early and late pregnancy, our comparative analysis shows a significant decline in HR MESOR and amplitude, alongside a significant increase in HRV MESOR and amplitude (Table 2). However, acrophase timing did not change significantly between early and late gestation, indicating consistency in peak timing despite changes in rhythm magnitude. Although prior studies have not systematically examined circadian rhythm parameters, our observed MESOR shifts-reflecting rhythm-adjusted mean values-align with trends of HR and HRV changes during pregnancy presented in the literature^34,51^.

In conclusion, our study reveals changing patterns of HR and HRV, characterized by circadian rhythm parameters, with a level of detail not previously explored. The use of wearable health monitoring devices for continuous, non-invasive, and cost-effective data collection, combined with machine learning techniques, enabled enhanced signal processing, data imputation, and analysis of circadian rhythms. Integrating these findings into prenatal care can optimize clinical decision-making and treatment strategies by enabling personalized interventions that account for the timing of physiological changes, ultimately improving maternal and fetal outcomes while lowering healthcare costs.

Although this study yielded significant findings, this study has several limitations that should be addressed in future research. First, participants were all white women, and the geographical location of the recruitment site (Turku, Finland) limited participant diversity. Also, the sample size (*n* = 30) provided limited data density, particularly during early (week 14) and late pregnancy (week 40), due to factors such as device familiarization period, late enrollment, and term deliveries before week 40.

Second, as mentioned in the introduction, both internal and external cues influence the circadian system. However, controlling for all of these factors is nearly impossible, and attempting to control even a subset may result in an overly restrictive living environment for participants. To address these challenges, we implemented specific inclusion and exclusion criteria aimed at minimizing certain confounding variables, such as excessive or insufficient physical activity and sleep. We also accounted for the presence of diseases and the use of medications known to affect the circadian system. However, we did not control for participants’ chronotype, work schedules, or distinguish between weekday and weekend patterns—factors that may reveal important variations in circadian rhythms due to daily routines and social timing (commonly referred to as social jet lag). In addition, participants’ diet and meal timing were not controlled.

Third, although respiratory behavior is known to influence HRV^52^, respiratory data were not collected in this study, as the Samsung Galaxy Watch model available at the time did not support respiratory rate monitoring. To mitigate potential confounding effects from respiratory sinus arrhythmia (RSA), we excluded participants engaged in interventions involving breathing techniques, such as square breathing. Furthermore, we selected RMSSD as our primary HRV metric, as it has been shown to be relatively less affected by respiratory influences compared to the HF component of HRV, making it more suitable for field studies where direct respiratory monitoring is not feasible. RMSSD has also demonstrated stability across a wide range of spontaneous breathing rates and is therefore widely adopted in ambulatory research settings^53^. Moreover, a recent study found no significant differences in nightly peak respiratory rates across the pre-pregnancy, pregnancy, and postpartum periods^51^. However, their analysis was limited to nighttime resting data and may not reflect respiratory variability or autonomic fluctuations that occur during daytime activities in free-living conditions. Although RMSSD is relatively robust to variations in spontaneous respiratory rate, it is not entirely immune. RSA can still introduce some variability in RMSSD^54^, particularly in cases of irregular or labored breathing. We acknowledge this limitation in our study and recommend that future research incorporate respiratory monitoring to further investigate the influence of respiration on HRV circadian parameters during pregnancy.

**Fig. 9:**
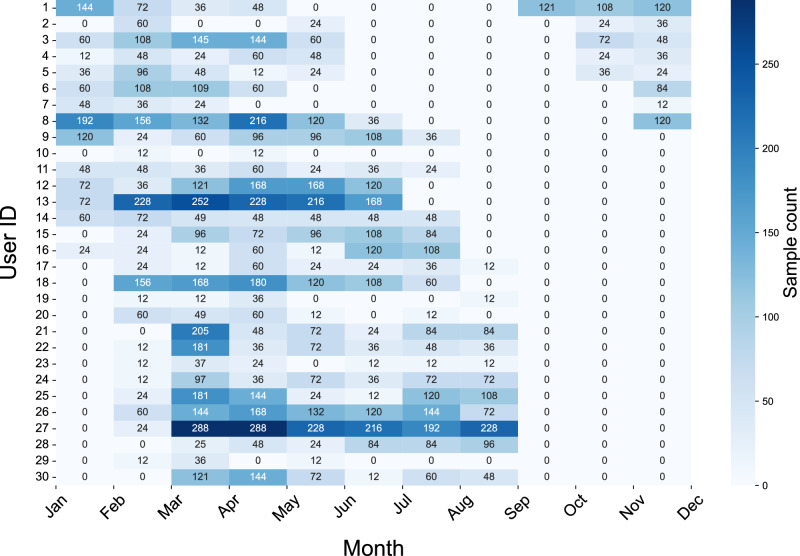
Monthly breakdown of samples containing both HR and HRV per user.

Fourth, participant recruitment occurred across different seasons, and the monthly breakdown of data density is presented in Fig. 9. Our study site experiences extreme seasonal daylight variability, ranging from approximately 6 h in winter to nearly 19 h in summer (worlddata.info/europe/finland/sunset.php). Since light exposure serves as a key photic cue for circadian rhythms, such variability likely induces maternal chronodisruption, potentially exacerbating pregnancy-associated sleep disturbances and consequently reducing HRV MESOR and amplitude, as well as altering acrophase. As shown in Fig. 9, most participant data collection began around January, when daylight was minimal (~6 hr/day), continued through late second and early third trimester during the peak daylight period (~17–19 h/day), and concluded in September, when daylight had decreased to ~13 h-coinciding with the end of pregnancy for most participants. Indeed, Fig. 5d reveals a pronounced acrophase delay, shifting from approximately 8 p.m. in early pregnancy to around 9–10 p.m., and in some weeks as late as 2–4 a.m. during extended-daylight summer months, which correspond to the late second and early third trimester for our participants. These delays are consistent with known effects of prolonged evening light exposure, which leads to phase delays and increased sleep irregularity^55^. To confirm these interpretations, future longitudinal studies are needed to objectively measure light exposure using ambient light sensors, allowing for a precise understanding of the impact of both sunlight and artificial light exposure duration and intensity on ANS functionality and sleep throughout pregnancy. Therefore, we acknowledge that these seasonal daylight variations may have influenced our results and could limit the applicability of our findings to settings with different lighting environments.

Fifth, while we acknowledge the limitation of not including a non-pregnant control group and recognize the value of such data for facilitating comparisons and better understanding pregnancy-specific changes, collecting longitudinal data from non-pregnant individuals may not yield accurate comparisons due to lifestyle and behavioral differences between pregnant women and non-pregnant women who are not actively planning pregnancy. Notably, 90% of our participants had planned their pregnancies and made preparatory lifestyle adjustments, which further complicates comparisons with general non-pregnant populations. Ideally, data collected from the same individuals prior to conception would provide the most reliable baseline for assessing pregnancy-induced physiological adaptations. However, due to the unpredictability of conception timing and the logistical challenges of recruiting participants before pregnancy, such data were not available at the time of this study.

Lastly, a methodological limitation of this study is the 20 Hz sampling frequency of the wearable devices used for HRV measurement. While this sampling rate is sufficient for reliable heart rate estimation, it offers limited temporal resolution (50 ms between samples), which may affect the accuracy of beat-to-beat interval detection. However, our HRV analysis focused solely on RMSSD, which is a time-domain metric relatively robust to low sampling rates due to its reliance on relative differences between successive intervals. In our prior validation study^56^, we demonstrated that RMSSD derived from our wearable devices sampled at 20 Hz showed high agreement with ECG-based measurements in both resting and free-living conditions. Furthermore, a key contribution of this work is the implementation of continuous, long-term monitoring using wearable devices in real-world environments, where higher sampling frequencies are often impractical due to hardware constraints and battery limitations.

While our study lays the groundwork for detecting abnormal circadian patterns in HR and HRV in pregnant women and their associations with pregnancy complications by first establishing normative patterns, future research should prioritize further investigation of HR and HRV circadian rhythms in women experiencing complications such as preterm birth and pre-eclampsia. Future endeavors focusing on identifying deviations in these rhythms among high-risk pregnancies, relative to the normative profiles established in our work, may offer early, noninvasive biomarkers for risk stratification and clinical decision-making in pregnancy complications. Ultimately, developing a machine learning model to predict pregnancy complications based on the identified biomarkers will be a critical next step toward enabling early intervention and improving maternal and fetal outcomes. Additional research directions include expanding these investigations to the pre- and post-partum periods, as well as exploring rhythm differences in pregnant women from diverse racial backgrounds and geographical locations with varying daylight exposure. Since circadian rhythms are closely linked with sleep^57^, and our findings indicate patterns of high stress levels during nighttime, which may both result from and contribute to sleep disturbances, considering sleep parameters could provide valuable insights into circadian changes throughout pregnancy.

## Methods

### Study design

We investigated the circadian rhythm of HR and HRV through a longitudinal health monitoring study on pregnant women^32^. This observational study was conducted under free-living conditions among pregnant women in Southwest Finland. In this study, participants were asked to wear Samsung Gear Sport smartwatches (samsung.com/us/mobile/wearables/smartwatches/gear-sport- black-sm-r600nzkaxar/) to continuously record HR and HRV, data during their daily activities. Additionally, a cross-platform mobile application was developed to gather subjective data such as Ecological Momentary Assessment (EMA) and background information, using self-report questionnaires. Data collection spanned from gestational weeks 12–15 and continued for three months postpartum.

### Participants and recruitment

Recruitment occurred from January 2019 to March 2020 in the Southwest Finland region. Recruitment was conducted via advertisements in maternity clinics and Finnish-language social media platforms. Interested women contacted the research team by email or phone, after which eligibility was assessed through direct communication.

Inclusion criteria were: (1) pregnant women with previous full-term births (i.e., after gestational week 37) and no history of pregnancy loss or complications; (2) age 18 years or older; (3) receiving regular prenatal care; (4) singleton pregnancy; (5) proficiency in the Finnish language; and (6) ownership of an Android or iOS smartphone.

Exclusion criteria included: (1) diagnosis of serious illnesses (e.g., cancer)^58^ or severe mental health conditions (e.g., clinical depression)^59^ that may affect circadian rhythms; (2) prescribed medical bed rest during pregnancy, due to reduced physical activity potentially disrupting circadian patterns^60^; (3) following an extreme diet^61^ or engaging in intense physical activity^60^; (4) diagnosis of severe sleep disorders (e.g., chronic insomnia)^62^; (5) regular use of medications or supplements known to influence sleep (e.g., melatonin)^63^; and (6) regular practice of structured breathing or relaxation techniques (e.g., square breathing, meditation involving breath control), which may significantly influence HRV patterns^52^.

Eligible participants attended face-to-face meetings with the researchers, where they were informed about the study objectives. Written informed consent was obtained, and participants received a smartwatch with detailed instructions to wear it continuously throughout pregnancy and for three months postpartum. Given the prospective, observational nature of the study and its free-living context, no lifestyle modifications were imposed.

We recruited 31 pregnant women with low-risk pregnancy profiles. One participant who developed gestational hypertension during the study was excluded from the analyses to prevent potential confounding effects. The remaining participants self-reported no pregnancy-related complications and were ambulatory throughout the study. Background information for the 30 participants is presented in Table 1.

### Research ethics

This study was approved by the Ethics Committee of the Hospital District of Southwest Finland (approval number Dnro: 1/1801/2018). All procedures involving human research participants were conducted in accordance with the relevant ethical standards of institutional and national regulations, as well as the 1964 Declaration of Helsinki and its later amendments. Written informed consent was obtained from all participants prior to their involvement, agreeing to the publication of results with assurances of data anonymization to safeguard their privacy.

### Data collection

HR and HRV data were collected from each participant using Samsung Gear Sport smartwatches (samsung.com/us/mobile/wearables/smartwatches/gear-sport-black-sm-r600nzkaxar/). These devices are equipped with an onboard photoplethysmography (PPG) sensor, enabling continuous physiological signal acquisition under free-living conditions. The smartwatch was selected based on several key features: access to raw PPG signals, programmable for developing custom data collection software, sufficient battery life for long-term monitoring, adequate internal memory, user engagement, comfort, and waterproofness.

A critical factor in our selection was the use of the open-source Tizen operating system, which allowed for the development and deployment of custom applications. These applications enabled fully automated, user-independent data collection and are compatible with other Tizen-based smartwatches. This system-level flexibility and hardware accessibility made the Samsung Gear Sport watch an optimal and affordable choice for our long-term wearable monitoring framework.

PPG signals were recorded for 12 min every 2 h at a sampling frequency of 20 Hz using customized applications installed on the smartwatches. This intermittent sampling approach was chosen to accommodate hardware limitations and optimize battery life, enabling continuous, long-term monitoring without requiring frequent recharging or user intervention. It allowed effective data collection over approximately 2 to 3 days with satisfactory battery performance. In our previous evaluation of the system design^32^, we found that continuous or frequent sampling (e.g., every 15 min) significantly reduced the battery life of the smartwatch to less than 24 h. In contrast, using a 2 h sampling interval extended battery life to 2–3 days, making it more practical for long-term monitoring in free-living conditions and reducing data loss during charging periods.

We developed a custom application to transfer recorded data from the smartwatch to a cloud server via a Wi-Fi connection. Although the smartwatch’s internal storage could hold data for up to two months, participants were instructed to upload their data at least once a week to ensure regular updates. On average, the PPG dataset used in this study included 111 min of recorded signal per day per participant.

### Data analysis

We utilized the collected longitudinal PPG data to analyze the circadian rhythm of HR and HRV during pregnancy. Our data analysis consists of three key steps illustrated in Supplementary Fig. 6. First, we employed a reliable machine learning-based PPG processing pipeline to extract accurate HR and HRV data from raw PPG signals. Second, we leveraged a machine learning approach to impute any missing HR and HRV samples in the data. Finally, we analyzed the circadian rhythm of HR and HRV during pregnancy using the Cosinor method.

HR and HRV Extraction: we extracted reliable HR and HRV parameters from longitudinal PPG signals using a PPG processing pipeline. PPG signals are highly susceptible to motion artifacts and noise, particularly when recorded in free-living conditions^64^. Corrupted PPG signals can lead to inaccurate HR and HRV measurements. To ensure the reliable extraction of these parameters from PPG, we employed a robust PPG processing pipeline, as proposed and evaluated in our previous work^65^. The pipeline implementation is publicly available as a Python package for reproducibility and broader use by the research community^66^.

The pipeline comprises multiple stages: filtering to remove unwanted frequencies, PPG signal quality assessment^67^ to identify high-quality signal segments, PPG reconstruction^68^ to correct minor noise and artifacts, and a robust PPG peak detection method^69^. Using this pipeline, we accurately extracted HR and HRV metrics, RMSSD, HF power, and pNN50, from 1 min PPG signal segments. The 1 min window length was chosen due to the high susceptibility of PPG signals to noise while ensuring reliable data extraction in shorter segments. HR and HRV values derived from the same 12 min signal segment were averaged for further analysis.

Missing Data Imputation: as our data collection was conducted in free-living conditions, it is common to encounter missing data due to the user’s hand movements or environmental noise. To address this issue, we developed a use-case-aware data imputation model to handle missing HR and HRV samples in our dataset. To ensure data quality, we included only days that contained HR/HRV values from at least seven different hours. Given our sampling interval of every 2 h (12 expected values per day), this criterion limited the number of missing values to a maximum of five per day. Across all participants, a total of 13,848 HR/HRV samples were expected over 1154 monitoring days. Among these, 5002 samples were missing, resulting in a missing sample rate of 36.12% and a valid acquisition rate of 63.88%. Supplementary Fig. 7a, b illustrate the distribution of missing samples by pregnancy week and participant ID, respectively. The variability observed reflects individual differences in lifestyle and activity levels.

We imputed the missing HR and HRV samples using a Long Short-Term Memory (LSTM) network, a type of Recurrent Neural Networks widely used for time-series data prediction^70^. LSTM networks capture and leverage temporal dependencies and patterns using memory cells that store information across time steps. These cells enable the network to preserve relevant information from previous samples, which is essential for predicting future samples in time-series data.

Our missing data imputation model consisted of three layers: one LSTM layer with 50 units and two dense layers. We trained and tested the model using sequential data created from available consecutive HR and HRV samples with a sequence length of six. The sequential data was split into training and testing sets, with 80% allocated for training. The model was trained over 100 epochs with a batch size of 5, using the Early Stopping technique to prevent overfitting. It is important to note that we normalized the sequential data by scaling it to a range of [0, 1] before training the model.

To avoid training bias and ensure generalizability, we split the sequential data into training and testing sets at the user level, ensuring that no data from a single participant appeared in both sets. This prevented user-level data leakage and allowed the model to be evaluated on entirely unseen individuals. Such an approach better reflects real-world deployment where missing data must be imputed for users not seen during training.

Cosinor Analysis of HR and HRV Circadian Rhythms: we employed Cosinor rhythmometry analysis^37^ to explore the circadian rhythms of HR and HRV during pregnancy. Cosinor analysis is a common method for studying rhythmic data and has been widely used for analyzing circadian rhythms^71–74^. This method involves fitting one or more cosine curves to rhythmic data using trigonometric regression models. A single-component Cosinor model is defined as follows:

1 $$Y(t)=M+A\cos \left(\frac{2\pi t}{24}+\phi \right)+e(t)$$

where M is the MESOR, A is the amplitude (half the distance between the peak and trough of the rhythm), the period is fixed to 24 h, *ϕ* is the acrophase (the time point in the cycle where the peak occurs), and *e*(*t*) is the error term. For multiple-component Cosinor analysis, which includes multiple cosine curves, the model is described by the following equation:

2 $$Y(t)=M+\mathop{\sum }\limits_{i=1}^{N}\left({A}_{i}\cos \left(\frac{2\pi t}{24}+{\phi }_{i}\right)\right)+e(t)$$

where *N* is the number of components (cosine curves) in the model, and each component has its amplitude *A*_i_, acrophase *ϕ*_i_, and a period of 24 h.

We applied the Cosinor model with different numbers of components (1, 2, and 3). The best-fitting model was selected using the extra sum-of-squares F-test, a statistical method for comparing the goodness of fit for models with varying complexity.

We employed a population-mean Cosinor approach to analyze the HR and HRV circadian rhythms during pregnancy. Within each week, we had multiple 24-h HR/HRV samples from a participant across several days. Our aim was to examine the weekly circadian rhythms for all participants throughout pregnancy. While the data from different participants were independent, the repeated measures within a week for the same participant were dependent. To address this structure, we applied the population-mean Cosinor analysis, as described in the literature^37,75^.

In this approach, a Cosinor model was first fitted to each participant’s daily 24-h HR and HRV data. Then, weekly averages for each participant were calculated by averaging the daily Cosinor results across several days of the week. Finally, the overall population rhythmic pattern for each week was determined by calculating the mean of the individual participants’ weekly averages.

To quantify the uncertainty around the fitted mean circadian rhythm, we computed and visualized the 95% confidence intervals of the population-level fitted rhythms as shaded gray areas in Figs. 4 and 7. Additionally, we calculated the residual SE for each weekly population-level Cosinor fit, which provides complementary insights into the systematic uncertainty and overall model performance, and is reported in the Supplementary Tables 1 and 2 for reference.

This same analytical approach was also applied to HF power and pNN50 measures.

We implemented our analysis methods using the CosinorPy Python package^75^, which is specifically designed for Cosinor-based rhythmometry. The analysis was conducted on a Windows machine equipped with an 11th Gen Intel Core^TM^ i7 processor and 16 GB of RAM.

To further evaluate trends in circadian regulation during pregnancy, we compared HR and HRV circadian rhythm parameters between early pregnancy (gestational weeks 14–20) and late pregnancy (weeks 34–40). Group-level means were calculated for MESOR, amplitude, and acrophase. Differences between early and late pregnancy were assessed, and effect sizes were calculated using Cohen’s d. A *p* value of <0.05 was considered statistically significant.

Correlation Analysis Between Maternal Age and HRV Circadian Rhythm Parameters: to explore the potential influence of maternal age on the circadian rhythm characteristics of HRV, we analyzed the correlation between participants’ age and the mean values of MESOR, amplitude, and acrophase of RMSSD across all pregnancy weeks. Pearson’s correlation coefficient (*r*) was reported, and a *p* value of <0.05 was considered statistically significant.

Leave-One-Out Cross-Validation (LOOCV) Analysis: To assess the robustness and stability of the estimated circadian rhythm parameters at the population level, we conducted a LOOCV analysis. For each gestational week, one participant was iteratively removed, and the population-level rhythm parameters-MESOR, amplitude, and acrophase-were re-estimated. We then calculated the mean and standard deviation of these parameters across all iterations to evaluate consistency. HRV was assessed using the RMSSD. Values are reported as mean (standard deviation) across gestational weeks.

## Supplementary information

Supplementary information

## Data Availability

The data utilized in this study include sensitive health information, and the informed consent signed by the participants strictly prohibits the public release of the data due to ethical constraints. According to the current approval by the Ethics Committee of the Hospital District of Southwest Finland, the participants explicitly granted permission to use the collected data solely for the purposes outlined in the consent agreement. Requests for data access require ethics committee approval. Researchers interested in accessing the data should reach out to the Principal Investigator of the research project, Associate Professor Anna Axelin, at the University of Turku, Department of Nursing Science, 20014 University of Turku, Finland; email: anmaax@utu.fi.

## Code availability

The developed code for this study is available from the corresponding author (M.R.) upon reasonable request.
