# Supplementary material for: Circadian rhythm of heart rate and heart rate variability in pregnancy
Source: NPJ Womens Health. 2025 Oct 7;3(1):57. doi: 10.1038/s44294-025-00107-6 (PMC12504112; doi:10.1038/s44294-025-00107-6)
Supplement: Supplementary file 1 — Supplementary information [file 44294_2025_107_MOESM1_ESM.pdf]

# Supplementary Information

Supplementary Table 1: **P-values and residual standard error (SE) of cosinor analysis results for heart rate across pregnancy weeks.** A p-value of <0.05 is considered statistically significant.

| Pregnancy week | P-value | Residual SE |
|---|---|---|
| 14 | 6.114e-13 | 7.44426 |
| 15 | 5.473e-11 | 9.10170 |
| 16 | 1.808e-09 | 9.02259 |
| 17 | 1.179e-12 | 9.71172 |
| 18 | 6.276e-08 | 9.70040 |
| 19 | 7.950e-07 | 8.49510 |
| 20 | 5.774e-08 | 9.66450 |
| 21 | 1.241e-08 | 9.02801 |
| 22 | 1.151e-07 | 8.18168 |
| 23 | 4.274e-14 | 7.82837 |
| 24 | 1.620e-06 | 9.63874 |
| 25 | 9.540e-06 | 9.38877 |
| 26 | 5.421e-10 | 8.55422 |
| 27 | 6.340e-03 | 9.40220 |
| 28 | 2.141e-11 | 8.35371 |
| 29 | 2.802e-13 | 8.48002 |
| 30 | 5.154e-04 | 9.64467 |
| 31 | 4.104e-03 | 9.28391 |
| 32 | 3.264e-05 | 9.56225 |
| 33 | 4.429e-07 | 8.95352 |
| 34 | 4.611e-07 | 9.63649 |
| 35 | 1.423e-07 | 9.43175 |
| 36 | 6.739e-05 | 10.21480 |
| 37 | 4.803e-07 | 10.12090 |
| 38 | 4.925e-09 | 9.56219 |
| 39 | 6.415e-08 | 9.07575 |
| 40 | 2.722e-02 | 11.33210 |

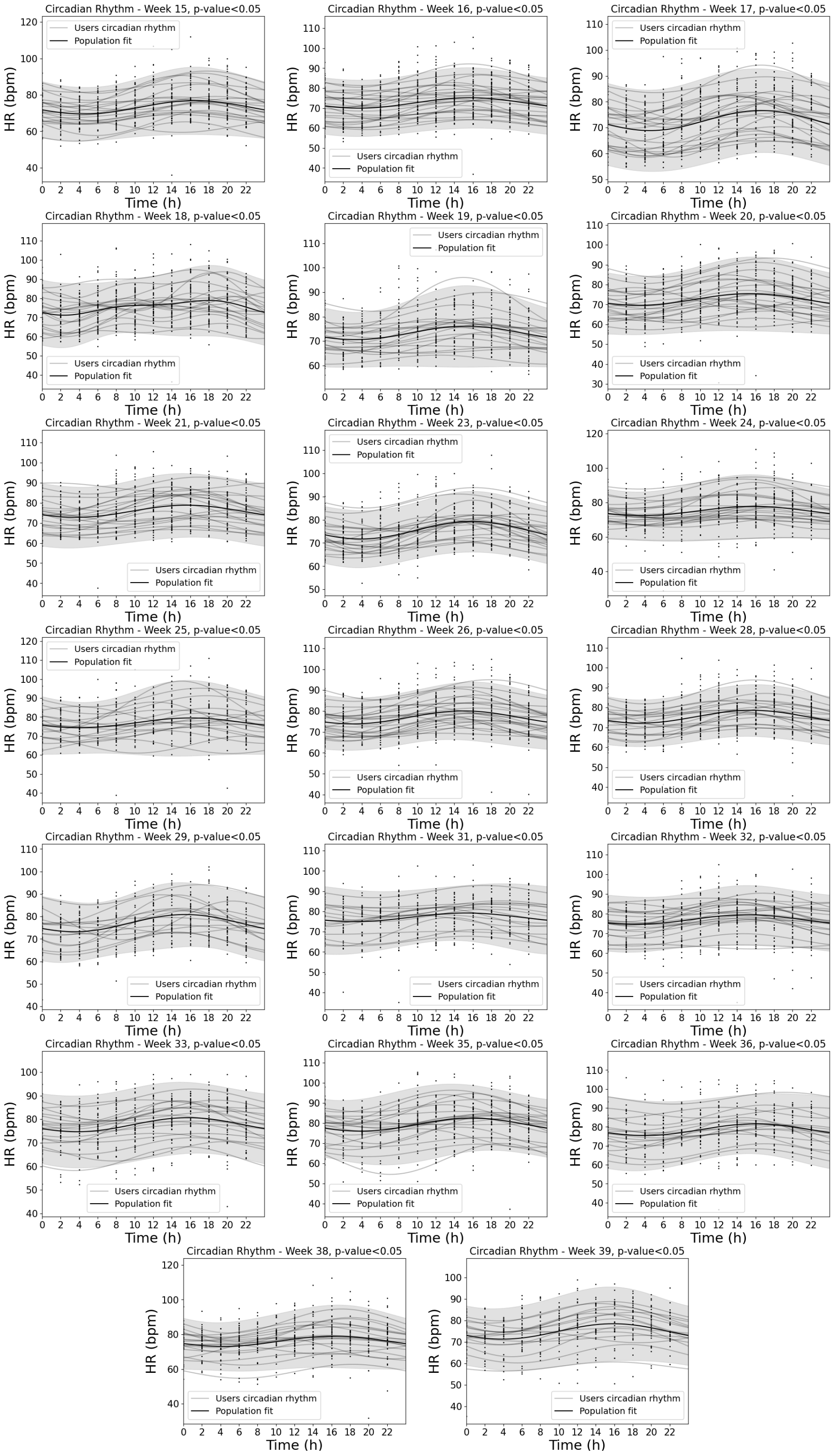


Supplementary Figure 1: **Cosinor analysis of the circadian rhythm of heart rate (HR) across pregnancy weeks.** Each subfigure shows the circadian pattern of HR for the corresponding pregnancy week. HR is reported in beats per minute (bpm), and time is reported in hours. Individual participants' weekly-averaged circadian data and rhythms are represented by black dots and gray lines, respectively, while the bold black line depicts the population-mean HR circadian rhythm. It should be noted that the number of data points (black dots) varies across weeks due to the unavailability of data from certain participants during specific weeks. A p-value of $<0.05$ is considered statistically significant.

Supplementary Table 2: **P-values and residual standard error (SE) of cosinor analysis results for heart rate variability (HRV) across pregnancy weeks.** HRV was measured using the root mean square of successive differences (RMSSD), a time-domain metric. A p-value of <0.05 is considered statistically significant.

| Pregnancy week | P-value | Residual SE |
|---|---|---|
| 14 | 2.877e-02 | 13.6930 |
| 15 | 4.676e-03 | 14.2673 |
| 16 | 8.890e-08 | 15.9637 |
| 17 | 1.744e-04 | 16.1930 |
| 18 | 1.169e-01 | 13.3061 |
| 19 | 1.319e-02 | 11.7260 |
| 20 | 3.384e-08 | 13.8330 |
| 21 | 1.588e-02 | 12.0654 |
| 22 | 4.457e-03 | 11.8701 |
| 23 | 7.255e-02 | 12.0572 |
| 24 | 5.660e-01 | 12.4676 |
| 25 | 2.064e-02 | 12.2170 |
| 26 | 5.725e-02 | 9.9858 |
| 27 | 1.685e-02 | 11.0260 |
| 28 | 4.588e-02 | 12.0219 |
| 29 | 3.070e-03 | 10.8204 |
| 30 | 9.193e-01 | 13.2850 |
| 31 | 4.425e-01 | 13.1811 |
| 32 | 3.057e-02 | 11.9664 |
| 33 | 4.726e-03 | 12.2469 |
| 34 | 8.587e-01 | 10.1941 |
| 35 | 2.673e-03 | 11.6447 |
| 36 | 1.572e-02 | 13.2084 |
| 37 | 5.675e-01 | 14.3159 |
| 38 | 3.825e-03 | 14.9659 |
| 39 | 1.064e-01 | 13.0005 |
| 40 | 1.055e-01 | 14.4046 |

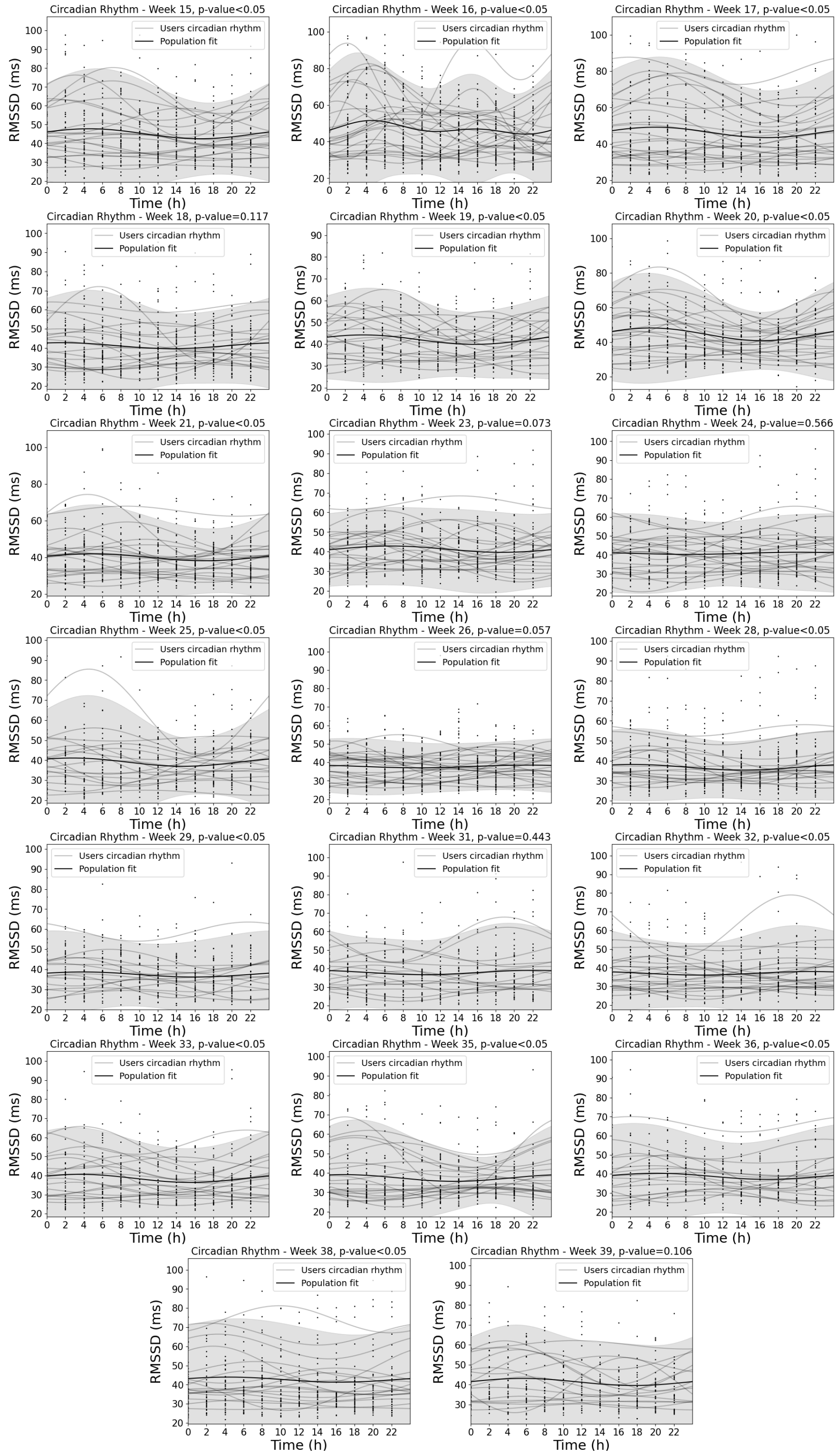


Supplementary Figure 2: **Cosinor analysis of the circadian rhythm of heart rate variability (HRV) across pregnancy weeks.** Each subfigure shows the circadian pattern of HRV for the corresponding pregnancy week. HRV was measured using the root mean square of successive differences (RMSSD), a time-domain metric reported in milliseconds (ms). Time is reported in hours. Individual participants' weekly-averaged circadian data and rhythms are represented by black dots and gray lines, respectively, while the bold black line depicts the population-mean HRV circadian rhythm. It should be noted that the number of data points (black dots) varies across weeks due to the unavailability of data from certain participants during specific weeks. A p-value of $<0.05$ is considered statistically significant.

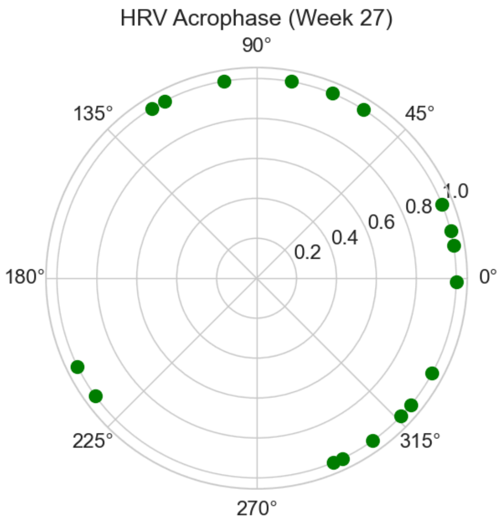


Supplementary Figure 3: **Individual heart rate variability (HRV) acrophase distribution in pregnancy week 27.** The polar plot shows the acrophase (peak timing) of each participant's HRV circadian rhythm fitted using Cosinor analysis. HRV was measured by the root mean square of successive differences (RMSSD), a time-domain measure.

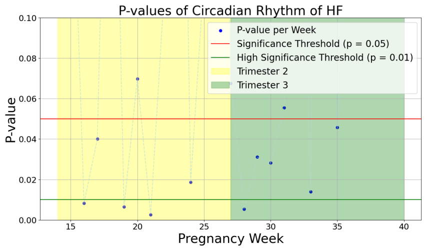


(a)

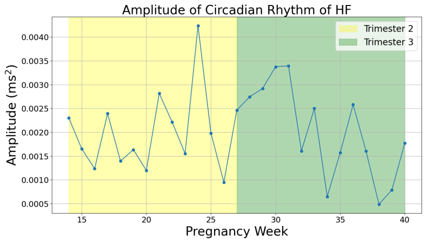


(b)

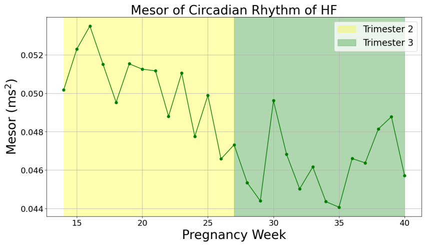


(c)

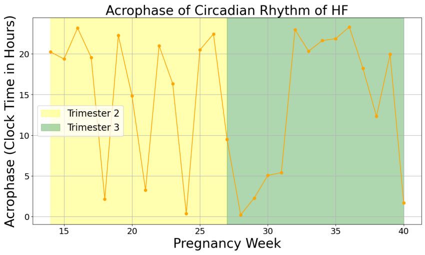


(d)

Supplementary Figure 4: **Cosinor analysis of heart rate variability (HRV) high-frequency power (HF) circadian rhythms during pregnancy.** Trends of (a) p-values, (b) amplitude, (c) midline estimating statistic of rhythm (MESOR), and (d) acrophase in the HF component, a frequency-domain measure of HRV, from gestational week 14 to 40. The MESOR and amplitude are reported in milliseconds squared (ms$^{\mathbf{2}}$), and acrophase is reported as clock time in hours. A p-value of $<0.05$ is considered statistically significant.

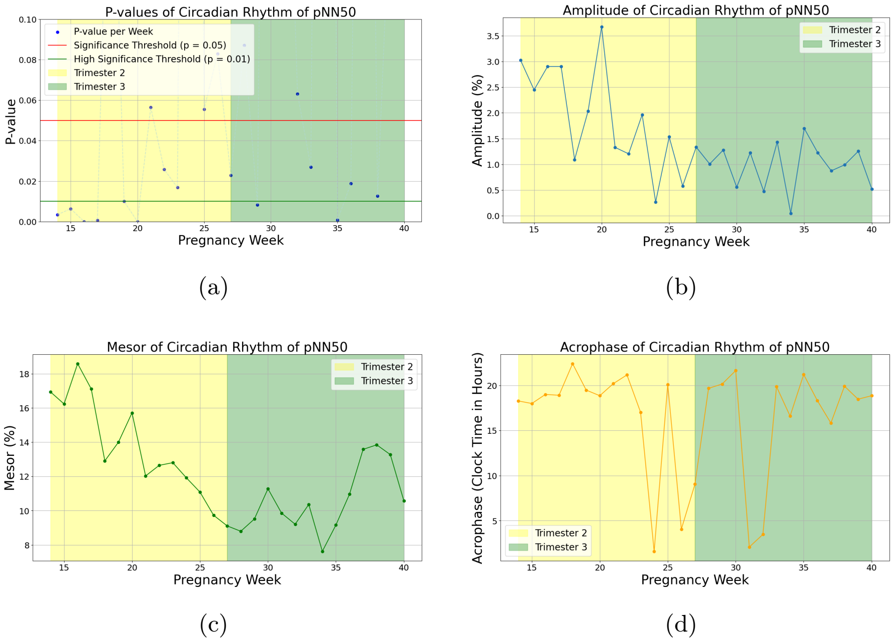


Supplementary Figure 5: **Cosinor analysis of heart rate variability (HRV) pNN50 measure circadian rhythms during pregnancy.** Trends of (a) p-values, (b) amplitude, (c) midline estimating statistic of rhythm (MESOR), and (d) acrophase in the pNN50 component, a time-domain measure of HRV, from gestational week 14 to 40. The MESOR and amplitude are reported as percentages (%), and acrophase is reported as clock time in hours. A p-value of <0.05 is considered statistically significant.

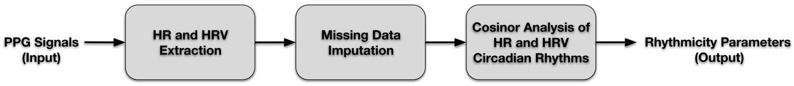


Supplementary Figure 6: **Data analysis workflow.**

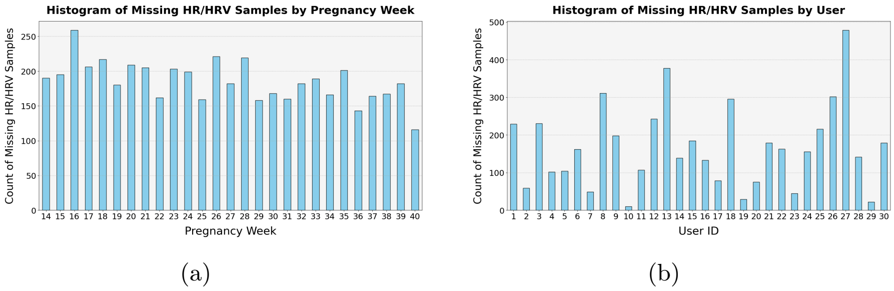


Supplementary Figure 7: **Histogram of missing heart rate (HR) or heart rate variability (HRV) samples in dataset by (a) pregnancy week and (b) user.**
